# Poor Mental Health and Its Impact on Governance Integrity in Developing Economies

**DOI:** 10.7759/cureus.89542

**Published:** 2025-08-07

**Authors:** Stanley Nkemjika, Colvette Brown, Ulunma N Umesi, Nkeiruka B Abanaka, Amarachi N Abanobi, Srikanta Banerjee

**Affiliations:** 1 Psychiatry, Interfaith Medical Center, Brooklyn, USA; 2 Environmental Health, Newton County Health Department, Covington, USA; 3 Population Health Sciences, School of Public Health, Georgia State University, Atlanta, USA; 4 Psychiatry, Yale School of Medicine, New Haven, USA; 5 Accident and Emergency, University of Calabar, Calabar, NGA; 6 Health Sciences, Northern Arizona University, Flagstaff, USA; 7 Health Sciences, Walden University, Minneapolis, USA

**Keywords:** developing economy, governance, integrity, mental health, social bias

## Abstract

Governance has traditionally been evaluated through political, economic, and institutional considerations, but new research indicates that bad mental health, and especially untreated anxiety, depression, and substance use disorders, can greatly undermine decision-making capabilities and institutional performance. Importantly, the perception of corruption among politicians and administrators has been described as being associated with heightened mental health-related manifestations. However, the direction of the associations between corruption and mental health is not well understood. Thus, there is a need to review and provide insight into the importance of integrating mental health into governance reform agendas in regions with high structural vulnerabilities. Therefore, this editorial spotlights mental illness as a crucial, though neglected, determinant of accountable, transparent governance, especially in developing economies, by probing the psyche at the roots of institutional behavior.

## Editorial

Poor mental health infrastructure could pose significant adverse outcomes on the integrity of governance, particularly in developing economies across Africa, Asia, the Caribbean, and Latin America. When adequate mental health care is not available, the fallout of unresolved psychological problems can reduce workforce efficiency, lower participation in community events, and upset social cohesion [1]. These long-term effects can undermine trust in the institutions and limit the ability of governments to provide important public services promptly and equitably. The connection between mental health and governance has not been thoroughly examined in current research, but there is significant potential for mental health problems to impact decision-making, institutional behavior, and even corruption [1,2]. Though studies have suggested relationships between corruption and mental health concerns [3,4], the direction of the causality remains elusive.

A survey of 730 participants in Ghana revealed that women, urban residents, moderately religious individuals, and those with lower national identification reported higher levels of perceived corruption [2]. To move beyond correlation, scholars can draw on theories from political psychology, behavioral economics, and institutional theory. For example, differential association theory and moral disengagement theory help explain how individuals under psychological strain may rationalize or adopt corrupt behavior in response to social norms [5]. From behavioral public administration, theories based on bounded rationality or cognitive load theory predict that chronic mental stress affects the ethical decision-making capacity of public officials [6]. Second, based on institutional theory, which highlights that weak rule of law and pervasive unaccountability in environments are breeding grounds for psychological disempowerment and unethical behavior, it suggests systemic governance failure, along with mental health outcomes [7]. Notably, the perception of corruption among politicians and government officials was most strongly linked to other corruption perceptions, and witnessing corruption in state institutions, alongside the belief that the wealthy can influence state actors, showed the most significant associations with depression and anxiety symptoms [2]. This highlights the need for robust, transparent infrastructure that promotes institutional accountability and reduces opportunities for elite capture. Strengthening governance mechanisms and ensuring equitable access to public services can help mitigate the psychological toll of perceived systemic injustice. Hence, investigating the relationship is important, considering that it may reveal more meaningful structural obstacles that developing economies need to overcome in order to attain accountable and transparent governance.

Governance, particularly in developing economies, is shaped by a complex interplay of political, economic, and social factors [8]. Central to effective governance is the ability of public officials and institutions to make decisions that advance economies that are geared toward the public good. However, the capacity for sound governance can be severely compromised when officials face untreated mental and behavioral health issues [9]. Mental health conditions such as substance use disorder, anxiety, and depression can undermine decision-making potential by impairing cognitive judgment, reducing emotional sensitivity, and reinforcing cognitive biases that may distort clarity and objectivity of the decision-making process [10]. Altruistically, the tenets of public health officials and institutions lie in their ability to make decisions that benefit the public good [11]. However, the effectiveness of governance can be significantly undermined when public officials struggle with untreated mental health or behavior-related conditions [9,12]. This could be particularly concerning with respect to corruption, where officials may be more vulnerable to unethical behavior due to their diminished ability to consider the long-term effects of their actions [13]. The link between mental health and corruption is particularly evident in countries where governance structures are fragile. Nations with unstable governance are often characterized by the absence of strong institutional oversight and accountability, which can lead to increased feelings of powerlessness and mistrust among citizens, triggering ongoing stress and mental health challenges. Continuous encounters with unjust systems and limited avenues for redress tend to intensify psychological strain, particularly for vulnerable groups who bear the brunt of corruption. In many developing countries, high rates of mental health issues among political leaders, civil servants, and other government officials may foster a culture of impunity and lower the commitment to ethical behavior [14]. Evidence in the literature noted that corruption thrives in environments where decision-makers are less able to think clearly and weigh the consequences of their actions [15]. Mental health issues like stress, trauma, and burnout, which are common in high-pressure political settings, may complicate these problems, which can lead to a decline in the integrity of governance [16].

Moreover, the stigma surrounding mental health globally, with a keen focus on many developing countries, can prevent individuals from seeking treatment, further exacerbating the cycle of poor mental health and poor governance [17]. In many African, Asian, Caribbean, and Latin American countries, mental health is often viewed through a cultural lens that may discourage open discussion or acknowledgment of psychological distress [17,18]. This cultural stigma can adversely affect both public officials and the rest of the populace from addressing mental health issues early on, causing them to deteriorate and result in a larger pattern of dysfunction in political institutions [18]. There is also a significant lack of mental health resources in these regions, which further compounds the problem. Developing economies often have limited mental health infrastructure, making it difficult for individuals in positions of power to access the necessary care [19]. Leaders and administrators in developing economies often overlook mental health, frequently viewing it as a taboo subject that receives little attention or support [19]. In fact, throughout the Caribbean and the diaspora, there is a shared belief that mental health illnesses are significantly attributed to a supernatural or spiritual force [20]. Given the sociocultural norms and attitudes around mental health, policymakers and individuals in position of influence may experience a unique mental health burden, and the stigma surrounding mental health poses a barrier to help-seeking behaviors [21].

The World Health Organization (WHO) has consistently reported that mental health services in low- and middle-income countries (LMICs) are underfunded, underdeveloped, and often stigmatized [22]. The lack of support or resources in LMICs may exacerbate the existing barriers experienced by public officials. Consequently, this increases their vulnerability to maladaptive coping mechanisms, such as corruption [23]. For example, evidence in the literature has suggested that weak health systems in LMICs are a major barrier to improving health outcomes, with structural adjustment programs (SAPs) imposed by international financial institutions since the 1980s being a key contributing factor [23]. These neoliberal policies, particularly in sub-Saharan Africa, led to reduced public sector spending, privatization, and deregulation, which destabilized public health systems through cuts in resources, the introduction of user fees, and workforce reductions [24]. The consequences of poor governance structures geared towards mental health systems extend beyond individual officials and permeate the entire political order [25]. Hence, mental health decompensation among a group of leaders responsible for making decisions has a systemic failure implication, as they may not execute their duties efficiently [26]. For instance, policy-making failures incurred as a result of stress may lead to the formation of situations that sustain corrupt activities [27]. This establishes a vicious cycle where the absence of proper governance encourages dishonest acts, which exacerbates the mental health burden problems of stakeholder officials.

Governance in developing economies is also complicated by the high levels of poverty and inequality that characterize many of these regions [28]. The social and economic pressures faced by individuals in leadership positions can contribute to mental health issues, which in turn may distort their behavior and decision-making processes [29]. The onset of high stress and mental health issues among these leaders may increase the likelihood of engagement in corruption for financial or self-serving benefits. Research has shown that individuals who are highly stressed tend to make decisions guided by their immediate needs, often at the expense of future benefits, which can lead to corrupt practices [30]. A study by Zhang (2022) revealed that higher corruption perceptions worsen depression, but trust in the government partially mediates the relationship [1]. This suggests that trust in government and patterns of media consumption may play a role in shaping the relationship between corruption perception and depression. Additionally, mental health conditions may engender lowered impulse control and greater emotional reactivity, which could translate to instances of violence, poor judgment, or unethical misconduct [31]. This may lead to a situation within government institutions where elected leaders prioritize their own interests over those of the people. Corruption becomes a coping mechanism for leaders who are unable to manage the emotional and psychological toll of their roles, especially in volatile and challenging political climates [1].

Is there a relationship between the level of corruption and the acceptability of mental health?

Evidence in the literature suggests that the lower the level of acceptability to accept mental health as a real issue, the higher the level of corruption within developing economies [2]. Most of these regions are highly corrupt, permeating both the public and private sectors, which creates a sense of impunity, a lack of transparency, and a fear of addressing issues deemed embarrassing, including mental health [2]. Political leaders and public officials in such environments are frequently under high levels of stress, yet acknowledging mental health challenges may be seen as undermining their authority or competence. In countries where corruption is endemic, mental health concerns are often dismissed as personal weaknesses, not deserving of public or institutional attention [32]. This bias may prevent mental health from being treated, which can further impair the ability of relevant stakeholders to make positive goal-driven decisions, which may then fuel further corruption and dysfunctional governance.

Furthermore, the stigma against mental health issues is made worse in these settings by the lack of mental health resources. In developing economies with high levels of corruption, funds that could be used in the development of public health, including mental health services, are wasted or misappropriated [33]. Not only does this limit the ability of those in power to receive appropriate treatment, but it also reinforces the widely held belief that mental health is of secondary importance [17]. Leaders who are unable or unwilling to address their own mental health struggles may be more susceptible to corrupt behavior due to impaired judgment, stress, and cognitive overload. Moreover, when working in the medical field, they are less likely to develop policies that promote mental well-being if they do not initially consider it a pressing matter [32,33]. However, societies that have made progress with curbing corruption tend to regard mental health issues as more important than their other counterparts. This is particularly in contexts where mental health issues are perceived as key to the success of leaders and their governance [17]. When political leaders recognize that they are psychologically affected and thus require assistance, they are better equipped to make informed business decisions that provide viable long-term solutions. A culture of openness and care can reduce stigma surrounding mental health and promote more accountable governance. Therefore, addressing mental health concerns and destigmatizing psychological care may contribute to reducing corruption in developing economies by improving the mental well-being of public officials and enhancing their ability to make sound, ethical decisions [34].

Role of social constructs and social determinants of health

The ideas and politics shape the level of integrity in health governance, and certainly, in most underdeveloped economies, people are more likely to engage in corrupt activities in unequal and poverty-pervasive areas [32,34]. Notably, ideas like gender, power relations, and cultural attitudes towards corruption can influence the way a person relates to such institutions. However, in many developing economies, such a corrupt political environment exists and is often expected, with social systems underpinning these structures. For example, in some societies, patronage networks are seen as legitimate means of accessing resources or political support, which can perpetuate corrupt practices [34]. Such constructs indeed undermine the moral standards of governance, as they not only encourage officials to steer away from a focus on rigorous accountability and transparency but also may be seen as incongruent with the prevailing social order. Furthermore, social factors such as poverty, education, and healthcare, among others, exacerbate the challenges in the fight against corruption [35]. In many developing economies, there are statistics of poor health and education, which are caked with extreme poverty that leads to mass corruption and bad governance [35]. Those individuals, especially leaders or citizens, who face serious emotional and physical health issues, tend to resolve such problems in a corrupt manner [35]. The circumstances that people have a difficult time navigating within the stratification system, combined with their lack of access to essential resources, may contribute to an increase in people's anger towards the government, low trust in the government, and a sense of disempowerment as citizens. This, in turn, encourages people to adopt the attitude that corruption is the only effective means of addressing systemic limitations [32,34,36]. Thus, striving to meet these social determinants could be essential in combating corruption, as society will be able to populate its members equally, always perceiving governance as an institution of social welfare rather than individual benefits [36].

Final thoughts

One key challenge in this area is the limited awareness and lack of research on the relationship between mental health and corruption. Although there are theories addressing mental health and social functioning, there is a shortage of studies that explore how weak mental health infrastructures, particularly in developing countries, may contribute to higher levels of corruption. Future research should investigate the causal pathways between inadequate mental health support for public officials and the normalization of corrupt behavior within state institutions. In this regard, policymakers and researchers need to adopt a more holistic approach to governance reform, one that considers the economic and political factors contributing to corruption, as well as the psychological well-being of those in positions of power. Focusing on the identification and mitigation strategies of public officials' mental health needs can significantly impact governance integrity. Public institutions and governments must take steps to ensure that mental health is prioritized as a key component of governance reform. Notably, proactive prevention strategies such as introducing mental health screening programs for stakeholders as pre- and post-recruitment criteria, implementing policies that foster mental well-being, and increasing funding for mental health services could help mitigate corruption in developing economic regions.

Furthermore, fostering a non-biased environment for openness and support regarding mental health is critical in overcoming the perceived bias that prevents individuals from seeking help [36]. These efforts may help reduce the cognitive and emotional impairments that contribute to corruption, thereby enhancing the integrity of governance systems. In addition to the above, however, creating a good climate to encourage people to talk freely and support each other about mental health is of paramount importance in fighting the stigma that would otherwise prevent people from seeking help. Working towards such goals should lead to a decrease in cognitive and emotional fetters that contribute to corruption, thereby improving the integrity of governance systems. An additional opportunity for enhancing this focus could be the integration of mental health awareness into anti-corruption initiatives. Investigating mental health issues within the context of corruption can provide a better perspective for anti-corruption programs aimed at understanding the causes behind unethical and wanton behavior. For instance, incorporating mental health training in the professional development of public officials as part of an anti-corruption initiative could be a vital feature in promoting integrity in governance. Therefore, it is undoubtedly advisable to include mental health as part of the close circle of reforms against corruption. Mental health help should not be regarded as a personal issue but rather as a key element for removing the political landscape fairly beyond overshadowing. The long-term benefits for public officials from good mental health may stem from increased psychological support for an effective governance system in any context of political behavior.

In conclusion, we suggest that mental health should be integrated and recognized in the context of governance and corruption, with the aim of improving defenses against corruption and maintaining institutional integrity, especially in developing economies. Therefore, more observational studies and healthcare policy-related research are greatly needed to highlight this public health issue. Mental health, which can be a hindrance to the abilities of public officers and leaders, puts them at risk of making decisions that may undermine the integrity of governance. We suggest that policies should include comprehensive reform approaches to incorporate mental health support for decision-makers, ensuring sufficient psychological resources are available for them to fulfill their duty of serving their people. This would be an overall public interest strategy to consolidate the welfare of public officers and leaders, aligning with the stated aim of achieving more transparent, accountable, and ethical political systems.
